# ETV2/ER71 Transcription Factor as a Therapeutic Vehicle for Cardiovascular Disease

**DOI:** 10.7150/thno.35300

**Published:** 2019-08-09

**Authors:** Dong Hun Lee, Tae Min Kim, Joo Kyung Kim, Changwon Park

**Affiliations:** 1 Department of Pediatrics; 2 Children's Heart Research and Outcomes Center; 3 Molecular and Systems Pharmacology Program; 4 Biochemistry, Cell Biology and Developmental Biology Program, Emory University School of Medicine, Atlanta, GA, USA; 5 Graduate School of International Agricultural Technology and Institute of Green-Bio Science and Technology, Seoul National University, 1447 Pyeongchang-daero, Pyeongchang, Gangwon-do 25354, South Korea

**Keywords:** ER71/ETV2, FLK1/VEGFR2, cardiovascular, angiogenesis, direct cell reprogramming

## Abstract

Cardiovascular diseases have long been the leading cause of mortality and morbidity in the United States as well as worldwide. Despite numerous efforts over the past few decades, the number of the patients with cardiovascular disease still remains high, thereby necessitating the development of novel therapeutic strategies equipped with a better understanding of the biology of the cardiovascular system. Recently, the ETS transcription factor, ETV2 (also known as ER71), has been recognized as a master regulator of the development of the cardiovascular system and plays an important role in pathophysiological angiogenesis and the endothelial cell reprogramming. Here, we discuss the detailed mechanisms underlying ETV2/ER71-regulated cardiovascular lineage development. In addition, recent reports on the novel functions of ETV2/ER71 in neovascularization and direct cell reprogramming are discussed with a focus on its therapeutic potential for cardiovascular diseases.

## Introduction

Transcription factors perform indispensable cellular events in living organisms because they can interpret genetic information in response to biological cues. The ETS transcription factors, consisting of approximately 28 members, are involved in diverse biological processes including cell cycle control, apoptosis (programmed cell death), embryogenesis, and tumorigenesis 1. The main distinguishing feature of ETS factors is the well conserved DNA binding domain (ETS DNA binding domain) that binds to the central sequences, 5'-GGAA/T-3', present on the promoters or enhancers of genes, leading to a wide range of biological consequences 1, 2. Besides the ETS DNA binding domain, the pointed domain (PTN) is shared among many ETS factors and mediates protein-protein interactions. Several members of the ETS factors such as ETS1/2, ETV2 (also known as ER71), FLI1, ELK3, and ETV6 are expressed in cardiovascular lineage cells in early stage embryos 3-11. In agreement with their expression pattern, these ETS factors have shown to be essential for the establishment and functions of the cardiovascular system constituents in the developing embryos 1, 4, 12.

Gene knockout studies have been instrumental for determining the biological consequences of genes *in vivo*. However, promiscuous binding specificity of the cognate binding sequences and the overlapped expression of the ETS factors often hinder the identification of the specific functions of individual ETS members. For example, *Ets1* deficiency in mice is compatible with the normal vascular development, but compound knockout of *Ets1* and *Ets2* leads to defects in vessel formation 13, 14. *Fli1^-/-^* mice succumb to death by embryonic day (E) 12.5 due to defective hematopoiesis and hemorrhaging in the brain 15. Since the overall embryo morphology including the cardiovascular structure of *Fli1*-null embryos is normal at E8.5-E9.5, it is likely that FLI1 is important for maintaining vessel integrity at mid-gestation. Germ line deletion of *Elk3* in mice develops defective vascular structures in adult retina 16. These findings suggest overlapped or mild functions of these factors in cardiovascular development. In contrast, our studies have shown that ETV2 is indispensable for the cardiovascular development during mouse embryogenesis 5. Our findings are consistent with the results from multiple groups working in diverse organisms such as zebrafish and *Xenopus* 17-21, suggesting that ETV2 is a critical regulator required for establishing the cardiovascular system in early embryogenesis. The first part of this review deals with the functions of ETV2 for cardiovascular system development. The potential applicability of ETV2 as a therapeutic agent in treating cardiovascular disease (CVD) will be discussed in the latter part.

## ETV2 is essential for the establishment of the cardiovascular system

In developing mouse embryos between E8.5 and E9.5, the expression of *Etv2* is preferentially detected in virtually all vascular structures including the dorsal aorta, cardinal vein, and endocardium. *Etv2* expression is downregulated at E10.5 and lost completely from E11.5 onwards 5, 9, 17, 18. In adults, the testes become the major organ for *Etv2* message while its expression remains low even in the highly vascularized organs such as the heart and lung 22. In addition, the first emerging FLK1 expressing (FLK1^+^) cells, which can subsequently differentiate into endothelial and hematopoietic cells 23, display enriched expression of *Etv2*, compared to FLK1^-^ cells as demonstrated by the results from both *in vivo* mouse embryos and *in vitro* mouse embryonic stem cell (mESC) differentiation system 5, 24. In agreement with the specific expression of ETV2 in hematopoietic and endothelial cell lineages in early stage embryos, gene knockdown and knockout studies also demonstrate the essential role of ETV2 in the cardiovascular system development. Such critical function of ETV2 was initially suggested from a study in zebrafish 25 by comparing the expression of genes between wild type and the zebrafish* cloche* mutant 26, characterized by the severe developmental defects in blood cells and endothelial lineages. The authors reported that *etsrp*, the zebrafish homologue of mammalian *Etv2*, was significantly downregulated in the mutant embryos, thereby suggesting functional significance of *etsrp* in blood cells and vessel development 25. Interestingly, zebrafish embryos injected with *etsrp* morpholino (MO) developed severely impaired vasculatures with a significant decrease of the expression of key markers of endothelial cells, but zebrafish embryos injected with *etsrp* mRNA showed ectopic expression of endothelial cell markers 20. Restored expression of *etsrp* in the *cloche* mutant embryos rescued the vascular defects. Similarly, the functional significance of *etsrp* in zebrafish vessel formation has also been reported by others 21. By performing N-ethyl-N-nitrosourea (ENU)-mediated mutagenesis and positional cloning, *y11* was identified as a mutant displaying defective embryonic vasculature, and *etsrp* was then identified as the gene responsible for these defective phenotypes. Ectopic expression of *fli1* and *flk1* was induced upon injection of *etsrp* mRNA into zebrafish embryos, but the significant reduction of endothelial and hematopoietic genes was observed in zebrafish embryos receiving *etsrp* MO. Subsequent studies in mouse have strengthened and expanded the findings from zebrafish. In 2008, we were the first to identify the essential function of ETV2 in cardiovascular system development in mice 5. *Etv2-*deficient mouse embryos displayed a complete lack of both hematopoietic and endothelial cell lineages, resulting in embryonic lethality between E9.5 and E10.5. Overexpression of *Etv2* in mESCs significantly increased the generation of cells expressing markers of hematopoietic and endothelial cells. These findings were further corroborated by the following studies from several independent groups; *Etv2* mutant mouse embryos generated through the gene trap approach died *in utero* and showed defects in embryonic vasculature development 17. Another report also demonstrated that *Etv2*-null mouse embryos failed to develop both cell lineages 18. Studies with mESCs have provided direct evidence of the determinant role of ETV2 in this process; *Etv2^-/-^* ESCs were incapable of generating hematopoietic and endothelial cells 27. The conserved importance of ETV2 in regulating endothelial cell development is also evident in *Xenopus* 19. It is of note that hematopoiesis appears normal in *Xenopus* embryos treated with *Etv2* MO. Collectively, these results suggest that ETV2 is essential for cardiovascular development.

## Target genes of ETV2 in vessel development

**Figure 1:**
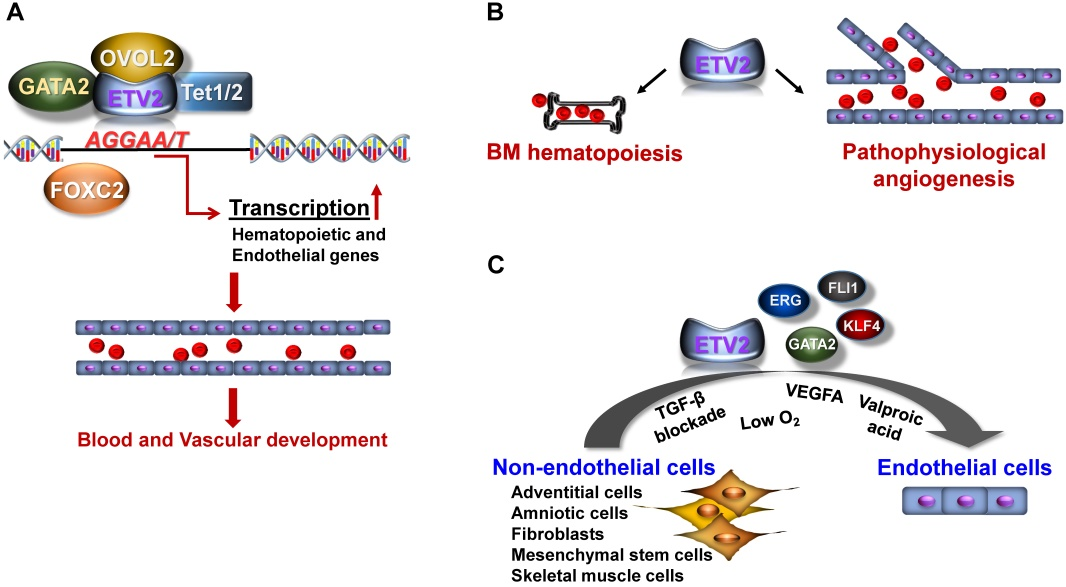
** Function of ETV2.** (**A**) In early embryogenesis, ETV2 plays indispensable roles for the establishment of the cardiovascular system through the direct binding to promoters or enhancers of genes critical for the hematopoietic and endothelial cell lineages. Recent reports suggest that ETV2 can form an active transcriptional complex at least with OVOL2, GATA2, FOXC2 or TET1/2. (**B**) In post-natal life, ETV2 regulates the proliferation of bone marrow (BM) hematopoietic stem cells. In addition, ETV2 robustly promotes new vessel formation in pathophysiological conditions such as injury, MI, and tumorigenesis. (**C**) ETV2 alone or in combination with other TFs or small molecules can directly reprogram non-endothelial cells into cells displaying endothelial functionalities.

In this section, we will mainly discuss the downstream targets of ETV2 that constitute a complex regulatory network for the development of embryonic vasculature. We will also discuss the mechanisms by which ETV2 activity is regulated (**Figure 1A**). For detailed discussion on the molecular mechanism of ETV2, the reader is directed to read the following reviews 28, 29.

As discussed, ETV2 is an ETS factor which can target genes containing the consensus sequences (5'-GGAA/T-3') present on enhancers or promoter regions 2. We were the first to report that ETV2 can directly bind to the *Flk1* promoter through the ETS binding elements and activate the expression of *Flk1* by performing the luciferase-based promoter assay and chromatin immunoprecipitation (ChIP)-PCR analysis 5. In subsequent studies, including ours, several key genes for vessel development and angiogenesis including *Tie2, Cdh5, Vegfr3, Robo4*, and* Notch4* have been identified as direct targets of ETV2 27, 30-32. Recently, a genome-wide analysis using ChIP-sequencing revealed that ETV2 has a wide range of direct downstream genes, further providing important insights into the molecular interaction between ETV2 and other signaling pathways 33. In this study, we found that ETV2 can directly activate genes closely associated with VEGF, Rho-GTPase and MAPK signaling as well as the aforementioned genes, all of which have been implicated in vessel development or hematopoiesis. Interestingly, ETV2 appears to activate its own expression and induces other ETS factors such as FLI1 and ERG 33. Considering the sequence specificity among the ETS factors, it is expected that other ETS factors can also bind some, if not all, of the ETV2 target genes and replace ETV2 function when ETV2 expression becomes silent 5, 9, 17, 18. Interestingly, the message of *Fli1* is rapidly induced by overexpression of ETV2, whereas *Fli1* deficiency does not affect the expression of ETV2 and the emergence of FLK1^+^ cells 33. A ChIP-PCR analysis revealed that day 4-5 embryoid bodies (EBs, cell aggregates of differentiating mESCs) expressing diminished level of *Etv2* showed enhanced *in vivo* binding of FLI1 on *Tie2*, *Cdh5* and *Gata2*, compared to day 3 EBs with high level expression of *Etv2* 33. *Fli1^-/-^* mESCs displayed a significantly reduced expression level of these genes, suggesting that FLI1 can, at least partly, inherit ETV2 function when ETV2 expression is depleted. This observation was further supported by another study 34 in which FLI1 failed to bind promoters of *Tie2* and *Cdh5*, when ETV2 is highly expressed. Interestingly, in the absence of *Etv2*, FLI1 is able to occupy these genes in mouse embryos. Moreover, *Tie2* and *Cdh5* expressions were reduced in *Fli1* knockout mice embryos, compared to control embryos. A similar relationship between ETV2 and FLI1 was also reported in zebrafish. Data from another group showed that inhibition of *etsrp* leads to severe vascular defects in zebrafish embryos, but a lack of both *fli1a* and *fli1b* is compatible with embryonic vessel development 35. Additionally, *etsrp* MO;*fli1b*-deficient embryos, not *etsrp* MO;*fli1a*- deficient embryos, displayed severe vascular defects, compared to *etsrp* MO embryos. The authors also demonstrated that *etsrp* and *fli1b* can redundantly induce the expression of the same target genes. Taken together, these results suggest that ETV2 initiates the cardiovascular developmental cascade by directly activating a wide range of genes in early embryos and later becomes silent after embryonic vessels have been established. In turn, other ETV2-induced ETS factors, such as FLI1, could partly replenish the vascular function of ETV2 by regulating subsets of the targets of ETV2 to mainly ensure the subsequent maturation and maintenance of vasculatures for successful embryogenesis. Sustained expression of ETV2 in mouse embryos leads to acquisition of endothelial character in non-endothelial organs 36. This supports the notion that ETV2 is short-lived, but critical for embryonic vessel formation 37.

Apart from blood vessel formation, it has also been suggested that ETV2 is involved in the development of lymphatic vasculature. In zebrafish, *etsrp* expression can be detected in cells fated to lymphatic vessels at 24-56 hours post fertilization (hpf) 38. Inhibition of *etsrp* by photoactivatable *etsrp* MO leads to defects in lymphatic vessel formation with impaired expression of lymphatic markers such as *vegfr3 (flt4)* and *lyve1b* 38. In agreement with these findings, the ChIP-sequencing assay and the promoter-based luciferase experiments reveal both *vegfr3* and *lyve1b* as direct downstream targets of *etsrp* 33, 38. Considering the evolutionarily conserved function of ETV2 and the increasing importance of the lymphatic vessels in pathophysiological events including CVD and cancer 39-43, a further detailed investigation on the role of ETV2 in lymphatic compartment in mammals would be warranted.

As other transcription factors, studies have shown that the activity of ETV2 can be regulated via its interacting proteins. Several endothelial genes including FOXC2 30, OVOL2 24, or GATA2 44 have been reported to interact with ETV2 in the generation of cardiovascular lineages (**Figure 1A**). Although detailed mechanisms of how these interactions control the functional activity of ETV2 need further investigations, the enhanced stability of ETV2 has been suggested as one of the mechanisms as demonstrated by the high level of ETV2 protein upon the interaction with OVOL2 24, 45, a zinc finger transcription factor known to play a critical role in angiogenesis 24, 45. Interestingly, a recent study found that ETV2 can also regulate the expression of its target genes through DNA methylation/demethylation by interacting with TET1/2 32, epigenetic modifiers responsible for DNA demethylation 46. The authors showed that ETV2 can directly bind the proximal promoter of *Robo4*, an important endothelial gene 47, through the ETS binding elements. Importantly, the interaction between ETV2 and TET proteins cooperatively potentiates DNA demethylation on *Robo4* proximal promoter, leading to the expression of *Robo4* in non-endothelial cells. Whether the expression of other endothelial genes can also be regulated by the interaction of ETV2 and TET1/2 is unclear. Nonetheless, this report suggests a novel insight into the mechanisms of ETV2 function in regulating its downstream target genes. Therefore, a comprehensive investigation to uncover the protein components of the ETV2 transcriptional complex would aid our current understanding of the functions of ETV2.

## ETV2 in pathophysiological angiogenesis and cell fate reprogramming

The potent role of ETV2 in vascular formation during embryo development led the investigators to explore the unknown functions of ETV2 in post-natal angiogenesis under the pathophysiological conditions. In the following sections, we will discuss recent findings from our laboratory and other groups regarding the therapeutic potential of ETV2 in mediating neovascularization and direct cell reprogramming (**Figure 1B-C**).

### Endothelial ETV2 is required for angiogenesis in response to pathophysiological stimuli

As discussed, ETV2 is specifically expressed in the vasculatures within a narrow developmental window between E8.5 and E10.5. In accordance with the transient expression pattern of ETV2, mice deficient in endothelial *Etv2* (i.e., *Tie2-Cre* or *Cdh5-Cre;Etv2^floxed/floxed^ mice*) are born alive and develop normal vascular structures 48, 49, suggesting that ETV2 is dispensable for steady-state vessel formation. There is a prevailing notion that embryonic events or signaling pathways become critical for the development of diseases or pathophysiological events in adults. This prompted us to investigate the function of ETV2 in post-natal life. Interestingly, we found rapid upregulation of *Etv2* in endothelial cells of mouse hindlimbs in response to ischemic injury 49, suggesting an important function of reactivated endothelial *Etv2* in injury-induced angiogenesis. By employing conditional knockout mice, we showed that the absence of *Etv2* in endothelial cells led to a significantly compromised vascular regeneration in response to injury such as eye injury, skin wounding, or hindlimb ischemic injury. Inversely, the overexpression of *Etv2* by injecting lentiviral *Etv2* into mice with ischemic injury facilitated recovery from the impaired blood perfusion and augmented the new vessel formation. Furthermore, the damage to tissues caused by the ischemic insult was repaired upon the injection of lentiviral *Etv2*. Mechanistically, ETV2 was able to activate critical genes for vessel development and angiogenesis, such as *Flk1, Cdh5* and* Vegfa/b/c,* as seen in developing embryos. Similarly, upregulated level of *Etv2* in tumor associated endothelial cells (TAECs) has also been reported in a previous study 48. The authors demonstrated that the deletion of *Etv2* in endothelial cells or systemic delivery of si*Etv2* into tumor bearing mice impairs tumorigenesis and angiogenesis. As ETV2 function is conserved in other vertebrates, *etsrp* expression in zebrafish, which is normally diminished by 2-4 dpf (days post fertilization) in developing embryos, is significantly induced not only in embryonic vasculatures, but also in tumor-associated vessels upon the transplantation of tumor cells 37. While wild type zebrafish injected with the tumor cells induced tumor-associated angiogenesis, blocking the function of *etsrp* using *etsrp* null mutant or photoactivatable *etsrp* MO led to severe impairment of the angiogenesis. Although beyond the vascular system itself, hematopoietic *Etv2* is also reactivated upon injury and plays an important function in bone marrow (BM) hematopoiesis (**Figure 1B** and also see next pages for further discussion) 50. Taken together, these results clearly suggest that reactivation of endothelial *Etv2* in response to pathophysiological stimuli is a central step for neovascularization and tissue repair (**Figure 1B**). In addition, the profound vascular regulatory functions of ETV2 *in vivo* upon delivery into mice in the form of lentiviral *Etv2* or si*Etv2*-nanoparticle complex raise the potential applicability of ETV2 as a therapeutic agent for diseases related to the dysfunctional vessel formation.

### Cell fate reprogramming by ETV2

ETV2 has also additional therapeutic potential owing to its critical role in the direct reprogramming of non-endothelial cells into endothelial cells (**Figure 1C**). Along with the groundbreaking iPSC generation from somatic cells 51, 52 with diverse methods, a significant effort has been made to convert one cell type into a distinct one by bypassing the pluripotent stage through the overexpression of cell lineage specific transcription factors 53-56 or miRNAs 57-60. The first successful demonstration of conversion of non-endothelial cells into functional endothelial cells was achieved in human amniotic cells (ACs) with a transient overexpression of *ETV2*, followed by the prolonged duration of other ETS factors, *ERG* and *FLI1* in conjunction with TGF-β signaling blockade 61. Transplantation of the converted endothelial cells into mouse models of neovascularization proves the functionality of the converted cells *in vivo*. It is important to note that ETV2 appears dispensable once ACs start expressing endothelial genes and that further maturation steps depend on ERG and FLI1, which is reminiscent of the sequence of events occurring in the developing embryos. In addition, it was shown that the battery of key endothelial transcription factors, ETV2, FLI1, GATA2, and KLF4, were able to generate cells with an endothelial phenotype directly from the human fibroblast 62. These results strongly suggest the feasibility of direct cell fate conversion for endothelial cell generation using the endothelial transcription factors. However, potential genetic burden caused by using a combination of multiple transcription factors and viral vector-mediated delivery is one of the major obstacles that needs to be overcome for clinical use of the reprogrammed cells. As a first step towards tackling the issue, Morita et al. reported direct conversion of the human dermal fibroblasts (HDFs) into endothelial-like cells using ETV2 alone 63. Delivery of the reprogrammed endothelial cells into mouse ischemic hindlimbs results in augmented perfusion with concomitant enhancement of neovascularization. The authors claimed that a transient expression of *ETV2* was sufficient for direct reprogramming. A modified method similar to this study demonstrated that culturing ETV2-transduced HDFs in hypoxic condition in the presence of VEGFA increased the efficiency of direct reprogramming of HDFs into cells expressing endothelial markers 64. We have also demonstrated a potent role for ETV2 in mediating the direct cell conversion process 65. In our study, we found two waves of the reprogramming process; a week-long overexpression of *ETV2* in HDFs induces so called early reprogrammed endothelial cells (rEC) characterized by substantial expression of key endothelial surface molecules including KDR and CDH5 with insignificant expression of mature endothelial markers such as CD31 and VWF. Despite the immature nature of early rECs, they are functional as evidenced by a significant enhancement of perfusion recovery from ischemic injury upon injection of the cells in a mouse model of hindlimb ischemia. The generation of endothelial cells displaying mature phenotype (i.e., late rECs) requires transient re-expression of ETV2 together with the treatment of an epigenetic modifier, valproic acid (VPA) during the cultivation of early rECs. Such treatments confer mature endothelial cell characteristics to the early rECs, such as high expression of CD31 and production of nitric oxide. In addition, genome wide RNA sequencing results show a close relation of late rECs to HUVECs or HUMVECs. Thus, our reprogramming protocol could be a novel system to study endothelial cell generation and maturation. Further, we believe that the early rECs could be useful as potential therapies and the late rECs would be a reliable source for drug testing. The function of ETV2 in mediating direct cell reprogramming into endothelial cells is further supported by studies in other species. For example, mouse adult skin fibroblasts can be directly reprogrammed into endothelial cells using a combination of ETV2 and other transcription factors including FOXO1, KLF2, TAL1, and LMO2 66. Mouse adventitial SCA1^+^ cells transduced with the adenoviral *Etv2* become cells displaying the endothelial functionalities *in vivo* and *in vitro* 67. In both studies, implantation of the directly converted cells in mouse vascular injury models leads to enhanced recovery from the injury by facilitating revascularization. In addition, a heat shock-mediated induction of *etsrp* in zebrafish embryos prior to 30 hpf can convert skeletal muscles into endothelial cells 68. Taken together, these results strongly suggest that ETV2 plays a critical function in direct cell conversion of non-endothelial somatic cells into endothelial cells, which could be used as cell therapy for CVD.

## ETV2 as a novel therapeutic agent for diseases with vascular defects

**Figure 2:**
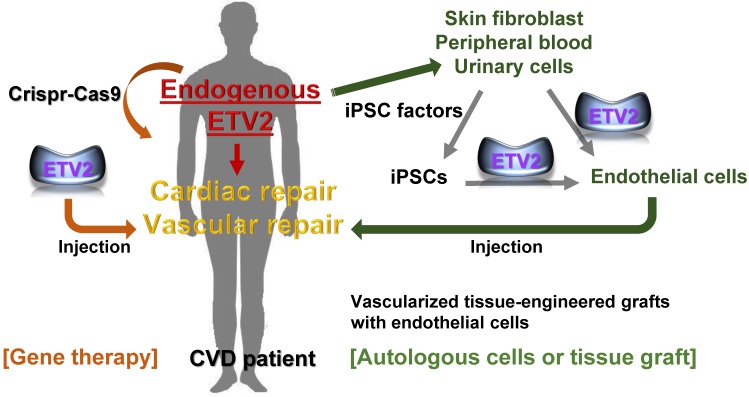
** The potential of therapeutic use of ETV2 in cardiovascular diseases.** Cardiac or vascular lesions caused by MI or PAD may be repaired by injecting ETV2, preferably in a non-genetic or non-viral form. In autologous cell transplantation model, reprogrammed endothelial cells can be obtained from non-endothelial cells including skin fibroblasts, peripheral blood or urinary cells via transient expression of ETV2 in these non-endothelial cells. Alternatively, iPSC-derived endothelial cells can be directly generated upon the overexpression of ETV2. Finally, vascular graft containing a patient's own reprogrammed endothelial cells would be an ideal option for repairing large vessels. Although far from being currently feasible, other approaches such as inducing endogenous ETV2 transcription machinery using the Crispr-Cas9 would provide an innovative strategy for vascular repair.

Dysfunctional blood vessel or uncontrolled vasculature formation is one of the leading factors that cause devastating diseases such as cardiovascular, cerebrovascular disease, age-related macular degeneration, post-operative complications after vascular surgeries, chronic wounds, and cancer. CVD ranks as the number one disease in the United States as well as worldwide 69, and treating patients with myocardial infarction (MI) and peripheral artery disease (PAD) remains one of the most profound challenges due to the diverse causes of these diseases. PAD can progress to critical limb ischemia, which causes a high incidence of amputation and mortality, necessitating efficient ways to treat the disease. Since the main cause of CVD is dysfunctional blood vessels resulting in loss or dysregulation of vasculatures, approaches for enhancing functional re-vascularization from the damaged tissues has gained extensive interest. For example, delivery of pro-angiogenic factors such as VEGFA, HGF, and FGF has been tested and found to have therapeutic benefits in animal systems 70-75. Mice undergoing ischemic MI show a long-term survival rate accompanying promoted myocardial function and enhanced angiogenesis upon reception of modified RNA form of VEGFA 76. Despite promising outcomes from animal or preclinical trials, the beneficial effects of pro-angiogenic approach are minimal at best in clinical trials with patients 77. This might be at least partly due to the intrinsic nature of the factors tested, most of which signal through their cognate receptor, limiting the width of angiogenic repertoires in patients who mostly have other comorbidities. Based on the basic developmental biology and recent progress in cell reprogramming, we understand that some lineage specific transcription factors dictate the expression of a wide range of downstream target genes, reshaping genetic and epigenetic status of cells. Studies have revealed that ETV2 can induce diverse endothelial genes through its DNA binding ability and its interacting proteins 5, 24, 30, 32, 33, 44, leading to strong vasculogenic and angiogenic events as well as cell fate reprogramming, acting as a master regulator of the vasculature formation and function. Hence, delivery of ETV2 alone or in combination with other pro-angiogenic factors would be a novel and efficient strategy for patients with MI or PAD (**Figure 2**). As a proof of principle, our group has already provided a promising data showing that ETV2 is involved in promoting vascular regeneration and tissue repair in a mouse model of hindlimb ischemia 49. Furthermore, injection of lentiviral or adeno-associated viral *Etv2* into mouse MI hearts enhances the recovery of heart function and myocardial angiogenesis 78. It is worthwhile to mention that ischemic hindlimbs that received lentiviral *Etv2* express reduced levels of inflammatory genes and develop a significantly lower degree of fibrosis 49. The same findings are also observed in mouse MI hearts upon injection of the lentiviral *Etv2* 78. Since unresolved or sustained inflammation may be detrimental and lead to chronic inflammatory diseases 79, these results further suggest the therapeutic advantages of ETV2 in reparative angiogenesis for various inflammatory vascular diseases.

In addition to inducing angiogenesis, inhibition of pathological angiogenesis or uncontrolled vessel formation can be a constructive means for preventing vascular-related diseases including tumorigenesis. As discussed, reactivation of *Etv2* occurs in TAECs, while interfering the function of ETV2 leads to decreased tumor angiogenesis in a mouse tumor model of xenotransplantation 48. Interestingly, a recent study demonstrates an augmented expression of *ETV2* in the patients of high grade of glioblastoma and reveals an inverse correlation between the expression level of *ETV2* and survival rate of patients of high grade of glioblastoma 80. Further, *ETV2* can stimulate the reprogramming of glioblastoma neural stem-like cells into cells with an endothelial phenotype. Additionally, deletion of *ETV2* in glioblastoma inhibits this process, suggesting novel functions of ETV2 in tumor-derived endothelial cell generation (i.e., vasculogenesis). Therefore, these findings suggest potent and broad spectrum of therapeutic potential of ETV2 in treating patients suffering from a diverse range of diseases including CAD, PAD and cancer.

Cell-based therapy is an additional method for treating ischemic vascular diseases (**Figure 2**). As discussed, upon overexpression of *ETV2* in HDFs, reprogrammed endothelial cells exhibit an endothelial phenotype and show augmented recovery of perfusion accompanying enhanced neovascularization in a mouse model of hindlimb ischemia. This is probably due to the paracrine effect and direct incorporation of the reprogrammed cells into the vessels of the ischemic tissues 65. In line with these findings, a recent study 81 reported that ETV2 can induce direct reprogramming of different somatic cells including human skeletal muscle cells (hSkMCs), adipose-derived mesenchymal stem cells, umbilical cord-derived mesenchymal stem cells, human embryonic lung fibroblast cells, and human skin fibroblast cells into endothelial like cells with hSkMCs being the best in terms of efficiency. In this study, the authors showed that co-culture of CDH5^+^ cells generated from *ETV2*-infected hSkMCs and skeletal muscle cells on PLGA/PLLA scaffolds or decellularized scaffolds leads to the formation of vascular-like structures in the engineered muscle tissues. By transplanting the engineered muscle tissue into athymic nude mice, they also demonstrated that the injected tissues survived and formed functional vascular network within the host as evidenced by the presence of red blood cells in the lumen. An additional study further expanded the applicability of ETV2 in direct cell reprogramming with human cell sources by demonstrating that a short period of ETV2 expression with inhibition of TGF-β signaling was able to generate cells with endothelial functionalities from human adipose-derived stem cells and human umbilical cord-derived mesenchymal stem cells 82. To make the current findings applicable to cell replacement therapy, direct reprogramming with patient-derived somatic cells would be an important prerequisite, taking into consideration immune compatibility. An additional obstacle that must be overcome is making the procedures for obtaining the cells as minimally invasive as possible. Peripheral blood and urinary cells would fit best as sources for reprogrammed endothelial cell generation (**Figure 2**).

ETV2 could also be exploited as a potential therapy by harnessing the biological traits of exosomes, extracellular vesicles ranging between 30-100 nm in diameter which contain proteins, mRNAs and miRNAs 83. The components of exosomes often reflect the pathophysiological status and the contents in exosomes are usually being transported to other cells. This biological function can be utilized for developing novel clinical therapies. Indeed, recent research in exosome biology has shown that exosomes derived from diverse sources can be used as biomarkers for certain diseases including CVDs and as therapeutics 84, 85. Regarding ETV2-exosomes, a recent study reported that CD31^+^ cells generated from lentiviral *ETV2* infected HDFs were able to produce exosomes 86. In a mouse model of hindlimb ischemia, the authors claimed that mice injected with the exosomes recovered efficiently from the ischemic damage, compared to mice injected with vehicles. However, further detailed analysis on the functions and mechanisms of the exosomes should be followed to firmly establish the potential role of the ETV2-exosomes in mediating neovascularization.

## Delivery and induction of ETV2 for therapeutic approaches

An issue that should be addressed is the fact that the observed beneficial effects of ETV2 were made possible with the viral particle delivery system, challenging the applicability of ETV2 for gene therapy or cell-based therapy for clinical use. Thus, a non-viral or even non-genetic delivery method for ETV2 *in vitro* and *in vivo* should be devised to eliminate the potential risk of the insertional mutagenesis caused by viral form of ETV2, resulting in chromosome instability. Success in cell reprogramming with modified mRNAs or chemical/small molecules 87, 88 has already been achieved. Therefore, a modified mRNA form of ETV2 or chemical/small molecules that can replace the lentiviral or adenoviral *ETV2* would be of particular interest from a clinical perspective. Generation of hematopoietic cells from human pluripotent stem cells (hPSC) by transfecting modified mRNA of ETV2 and GATA2 has been reported 89. The same group has also shown that modified mRNA of ETV2 alone is capable of inducing cells displaying an endothelial phenotype from multiple lines of hPSCs and non-human primate iPSCs 90, raising the feasibility of ETV2 as a therapeutic agent. Specific induction of endogenous ETV2 with a Crispr-Cas9 approach (e.g., delivery of sgRNA specific to endogenous *ETV2* promoter/ enhancer and dCas9-VP64) would be another option to pursue 91-93. In addition, efforts should be made for the development of targeted delivery methods of modified RNA form of ETV2, siRNAs or small molecules to allow the clinical potential of ETV2 to be realized.

It would also be important to identify upstream signaling that can induce the expression of ETV2 in the vascularization as this may be of significance for therapeutic purposes. In this regard, we have previously reported that ETV2 functions downstream of BMP, WNT and NOTCH signaling in generating cardiovascular lineages from mESCs 5. Additionally, NKX2-5, foxc1a/fox1b, MESP1-CREB, and IP_3_Rs-Ca^2+^-CALCINEURIN-NFATc3 17, 94-97 have been identified as direct upstream regulators of ETV2. Among them, we will focus on two important signaling events, calcium signaling and reactive oxygen species (ROS). Recent studies have shown that calcium (Ca^2+^) signaling plays a critical role in *Etv2* expression. Sequence analysis on the *Etv2* gene locus revealed two conserved cAMP response element (CRE) in the promoter and 5'-untranslated region (UTR). Mechanistically, the cAMP/protein kinase A (PKA) pathway can induce *Etv2* expression via direct binding of CRE binding protein (CREB) to the CRE located in the 5'-UTR of *Etv2* 97. Since Ca^2+^ can activate adenylyl cyclases which are responsible for the conversion of ATP into cAMP 98, these findings suggest that intracellular Ca^2+^ influx can act as an important upstream inducer of ETV2 expression via cAMP-PKA-CREB signaling. Another study demonstrated that *Mesp1*, a bHLH family transcription factor for cardiac mesoderm specification 99, can activate transcription of *Etv2* by the MESP1-CREB interaction in CRE within the *Etv2* 5'-UTR 95. More recently, it was shown that NFATc3, an effector DNA-binding protein downstream of the Ca^2+^/ CALCINEURIN pathway, occupies an important position in promoting hematopoietic lineage commitment from FLK1^+^ mesoderm by direct binding to the *Etv2* promoter 96. Blocking Ca^2+^/CALCINEURIN signaling in differentiating mESCs by deleting inositol 1,4,5-trisphosphate receptors (IP_3_Rs), which are the major calcium releasing channels in endoplasmic reticulum, or by treating CALCINEURIN inhibitors, led to a decrease in *Etv2* expression. However, treatment of ionomycin, or overexpression of a constitutively active form of CALCINEURIN or NFATc3 was able to induce the expression of *Etv2* in wild type and *IP_3_Rs* deficient mESCs. Further, it was shown that NFATc3 can directly occupy the NFAT and NFκB binding sites in the *Etv2* upstream region *in vivo* 96. Taken together, these findings suggest that identifying bioactive molecules that can regulate Ca2^+^ related signaling seems to be an effective option that can induce the expression of ETV2 in mediating vascularization in adults. One candidate would be adrenomedullin, a vasodilatory hormone peptide, which has been shown to exhibit various effects on vessels such as inhibiting endothelial cell apoptosis and promoting vessel growth through several important signaling pathways including Ca2^+^ and cAMP 100, 101. Importantly, administration or overexpression of adrenomedullin promoted (lymph)angiogenesis, while reduced expression of adrenomedullin led to impairment of vessel recovery in murine models of vascular injury 102-106. At least in differentiating mESC, adrenomedullin was able to increase the generation of ETV2^+^ cells and the expression of *Etv2* 97. The functional relation between adrenomedullin and ETV2 in pathophysiological angiogenesis in adults needs further investigations.

Another fundamental approach on *Etv2* induction have focused on ROS, whose function is also involved in promoting physiological angiogenesis if properly controlled 107. Upon hematopoietic cell injury by 5-FU injection, the expression of hematopoietic *Etv2* and the ROS production increases in hematopoietic stem and progenitor cells (HSPCs) as defined by c-KIT^+^Sca1^+^Lin^-^ 50. Remarkably, the expression of *Etv2* was augmented in HPSCs after being treated with an ROS activator, whereas 5-FU induced augmentation of *Etv*2 expression was abrogated in the presence of a ROS scavenger and an NADPH oxidase inhibitor, suggesting that ROS function as a critical player in *Etv2* activation 50. More direct evidence on ROS-mediated *Etv2* induction was shown in TAECs from lung carcinoma in a tumor graft mice model. A higher level of ROS was detected in TAECs than in endothelial cells from the normal lung or hindlimb tissues. Treatment of an ROS scavenger or siRNA against *Etv2 in vivo* resulted in down-regulation of the expression of *Etv2* in TAEC, and led to reduction of the tumor volume 48. These results indicate that ROS functions as an important inducer of *Etv2*. Collectively, these studies present a novel direction for the development of therapeutic strategies by bringing Ca^2+^-related signaling, ROS, other intermediate signaling molecules to the territory of ETV2 in vascular disease.

## Conclusions

ETV2 has increasingly gained attention due to its potent vasculo-angiogenic properties. Over the past decade, studies have revealed the important functions of ETV2 and its underlying mechanisms. However, our current knowledge about ETV2 is still too limited to make this molecule therapeutically feasible. The advent of new research tools such as the next generation sequencing, Crispr-Cas9, epitranscriptomics and biomaterials will facilitate understanding of the mechanisms and functions of ETV2. This, in turn, could aid the development of safe and novel ETV2 therapeutic options to treat diseases related to vessel dysfunction. As discussed above, preclinical testing of ETV2 in inflammatory vascular diseases (e.g., atherosclerosis) would also be important since vascular diseases are often accompanied with chronic inflammation. Lastly, it is important to note that the therapeutic applicability of ETV2 can be expanded to the lymphatic vascular system. Experimental results from several animal models including rabbit and dog suggest a close association of impaired lymphatic flow and CVD 39, 108-110. Moreover, etsrp plays an important function in regulating lymphatic vessel formation in zebrafish 38. Thus, further detailed studies regarding ETV2-lymphatic vessel/disease would be an interesting area to pursue for basic research and translational purposes.

Although rodent models have significantly contributed to the understanding of early vessel development and pathophysiology of various CVDs, it should also be noted that their cardiovascular physiology and inflammatory profiles are different from humans 111, 112. Accordingly, large animal models are often preferred because they are accepted as more clinically relevant models of human physiology, despite the limited number of available reports 113. In a sheep model of MI, when injected directly into the myocardium one hour after coronary artery ligation with a plasmid DNA expressing human VEGFA_165_ under CMV promoter/enhancer, the resting myocardial perfusion was increased while the infarct area was reduced 114. Additionally, the capillary density was higher in the VEGFA_165_-treated animals than in placebo group. More recently, another study in a swine model of MI reported that adenovirus carrying human SCF enhanced cardiac functions, with an increase of c-KIT^+^ cells, vessel density, and a decrease in apoptosis 115. In a dog model of hindlimb ischemia, human bone marrow- derived mesenchymal stem cells together with the sustained release of bFGF via heparin-conjugated fibrin promoted the angiogenic effect, as indicated by the increased arterioles and capillaries, as well as PDGF- and VEGFA-positive cells 6 months after transplantation 116. Large animal models are essential, not only for their usefulness in translating preclinical results into clinical research, but also for determining optimal dosage and delivery route for human use 117. Together with well-developed interventional approaches, the use of large animal models with a special focus on ETV2 will be instrumental for confirming the initial findings from rodent studies, and finally will significantly contribute to the development of novel gene- or cell-based therapeutics for CVDs.

## Abbreviations

ACs: amniotic cells
BMP: bone Morphogenetic Protein
CAD: coronary artery disease
c-AMP: cyclic adenosine monophosphate
CAS9: CRISPR associated protein 9
CD31: cluster of differentiation 31 (also known as PECAM1)
CDH5: cadherin 5 (also known as VEcadherin)
c-KIT: receptor tyrosine kinase (also known as stem cell factor receptor, CD117)
CREB: c-AMP response element binding protein
CLI: critical limb ischemia
CMV: cytomegalovirus
Crispr: clustered regularly interspaced short palindromic repeats
CVD: cardiovascular disease
E: embryonic day
EB: embryoid body
ELK3: ETS domain-containing protein
ERG: ETS-related gene
ESCs: embryonic stem cells
ETS: E26-AMV virus oncogene cellular homolog, a transcription factor
ETV2: ETS Variant 2 (also known as ER71 or etsrp)
ETV6: ETS Variant 6
FGF: fibroblast growth factor
FLI1: friend leukemia virus integration 1
FOXO1: forkhead box O1
5-FU: 5-fluorouracil
GATA2: GATA-binding factor 2
HDF: human dermal fibroblast
HGF: hepatocyte growth factor
HSPCs: hematopoietic stem and progenitor cells
HSFs: human skin fibroblast cells
hSkMCs: human skeletal muscle cells
IP3R: inositol trisphosphate receptor
iPSCs: induced pluripotent stem cells
KDR: kinase insert domain receptor (also known as FLK1 or VEGFR2)
KLF2/4: kruppel like factor 2/4
LMO2: lim domain only 2
MAPK: mitogen activated protein kinase
MESP1: mesoderm posterior protein 1
MI: myocardial infarction
MO: morpholino
NFATc3: nuclear factor of activated T cells 3
NKX2-5: NK2 homeobox 5
NO: nitric oxide
NOTCH4: neurogenic locus notch homolog protein 4
PAD: peripheral artery disease
PDGF: platelet-derived growth factor
PI3K: Phosphoinositide 3-kinase
PKB: Protein kinase B
PLLA: polylactic acid
PLGA: poly lactic-co-glycolic acid
PSC: pluripotent stem cell
Rho-GTPase: ras homolog gene family GTPase activating protein
ROS: reactive oxygen species
SCA1: stem cells antigen1
SCF: stem cell factor
sgRNA: single guide RNA
TAL1: T-Cell acute lymphocytic leukemia protein 1 (also known as SCL)
TET1/2: ten-eleven translocation ½
TGF: transforming growth factor
TIE2: TEK tyrosine kinase
VEGF: vascular endothelial growth factor
VEGFR3: vascular endothelial growth factor receptor 3
VWF: von willebrand factor
VPA: valproic acid
WNT: wingless-related integration site
